# Strengthening US Public Health Preparedness and Response Operations

**DOI:** 10.1089/hs.2016.0115

**Published:** 2017-02-01

**Authors:** Matthew Watson, Jennifer B. Nuzzo, Matthew P. Shearer, Diane Meyer

In the years following the 9/11 attacks, the leadership, resources, and expertise that public health agencies across the country can bring to bear on the response to infectious disease emergencies and other catastrophic events took on additional urgency and importance. In light of this expanded mission, Congress appropriated funds to support public health preparedness at the state and local levels, one result of which was the Centers for Disease Control and Prevention (CDC) Public Health Emergency Preparedness (PHEP) program. Several recent infectious disease emergencies—chief among them the Ebola epidemic in West Africa and isolated cases in the United States (2014-15) and the ongoing Zika virus pandemic—have challenged national health security and demonstrated a need for continued investment in domestic public health preparedness and response infrastructure.

## Recommendations

➢ **Provide increased funding for the Public Health Emergency Preparedness program.**

Federal support remains vital to national preparedness for a range of intentional, accidental, or naturally occurring epidemics, and real gains have been made since 2001. The CDC reports that all or nearly all health departments in the country are currently able to mobilize staff to respond to an emergency, collaborate with healthcare providers for the purpose of preparedness planning, and distribute medical countermeasures (ie, the therapeutics and vaccines needed during an emergency), which was not the case in the pre–9/11 era.

Declining federal support for public health preparedness, however, puts these gains in jeopardy. Specifically, funding for PHEP has decreased by 40% from its peak in FY2006.^1,2^ The effects of these cuts are being felt nationwide, as health departments have been forced to make significant reductions in staff and preparedness activities. According to the National Association of County and City Health Officials, more than 51,000 jobs have been lost at local health departments since 2008.^3^ These cuts translate directly to a decreased ability to mount a timely, effective response to routine and extraordinary threats, including infectious diseases, natural disasters, and terrorist incidents. To ensure the maintenance of key public health preparedness infrastructure, funding for this program should be restored to FY2006 levels, or approximately $1 billion per year.

While preparedness has been historically difficult to measure in absolute terms, tools like the recently developed National Health Security Preparedness Index can be used to assess progress in this area and potentially to help guide resource allocation decisions. Ultimately however, in an increasingly constrained financial environment, federal funds for preparedness should be prioritized for those localities judged to be at highest risk.

➢ **Ensure support for the provision of clinical care for infectious diseases.**

Public health clinics contribute significantly to the nation's outbreak response capabilities and should be supported as a matter of priority. At many health departments, clinical staff that typically provide care for patients with routine conditions, such as tuberculosis and sexually transmitted infections, are important sources of surge capacity for responding to public health emergencies, such as those posed by Zika, Ebola, MERS, chikungunya, and dengue. Public health clinicians who work to interrupt disease transmission on a daily basis have the necessary skills and experience to lead or support disease control activities like outbreak investigations, mass vaccination campaigns, public education, and others. For example, public health clinicians who work on reproductive health in their daily duties formed teams that went door to door in Miami, Florida, to conduct surveillance for Zika virus infection. But budgetary constraints have forced many health departments to scale back or shutter public health clinics where these vital personnel operate.

As discussions about the future of the Affordable Care Act progress, the Administration should work with Congress to ensure that future healthcare legislation enables patients with infectious diseases that pose a significant threat to the public's health to have access to care. It is critical that cost concerns not stand as a barrier to care for patients with communicable diseases. Cost barriers such as high deductibles and copayments can preclude even those covered by health insurance from seeking care, prolonging the course of their disease and increasing the likelihood of community transmission.

Services for these diseases—including screening, diagnosis, treatment, and case management—should be covered without cost-sharing for patients, regardless of their insurance status or where they seek care. For those insured patients who seek care at a public health clinic, that health department should be permitted and able to submit for reimbursement to ensure that they can continue providing such services. The identification and treatment of patients with infectious diseases is critical to interrupting transmission, and ensuring access to care is essential for the protection of the public's health.

➢ **Establish a public health emergency fund.**

Once an event has triggered a local, regional, or national public health emergency, health departments shift from preparedness to response activities. While the specific response activities implemented by health departments depend on context, examples include the ability to rapidly vaccinate or distribute medications to a given population, conduct outbreak investigations and surveillance, and communicate risk information to the public.

In addition to sustained funding to maintain the core public health infrastructure that supports preparedness programs, additional funding is needed during the response to public health emergencies. Public health authorities have historically depended on the provision of an emergency appropriation by Congress to finance the response to infectious diseases like H1N1 influenza and Ebola; however, this reliance on emergency appropriations can lead to dangerous delays. Delays in the availability of funding created significant challenges in responding to Zika infections occurring in the United States. In Florida, which had seen local transmission of Zika, patients were forced to wait for weeks for their test results because of insufficient laboratory capacity in the state. Now that an emergency appropriation has been passed, the challenges caused by a delayed response are not likely to abate in short order, as health officials have said that it may be several more months before states receive federal funding.

To ensure that public health can respond effectively and in a timely manner to future health crises, Congress should enact a modern Public Health Emergency Fund. Based on past emergency appropriations, our own analysis, and that of expert advisory groups such as the President's Council of Advisors on Science and Technology, we recommend an appropriation in the amount of $1-2 billion. Such a fund should be maintained separately from federal preparedness grants, be made available immediately upon declaration of a public health emergency, and be of a magnitude sufficient to jumpstart a nationwide response to a recognized threat.

➢ **Build an emergency medical team for use during epidemics.**

Finally, the Administration should direct the development of a deployable team capable of providing clinical care surge capacity for use during epidemics and disasters. The West African Ebola epidemic clearly demonstrated that the nation lacks the ability to rapidly support the provision of clinical care in an emergency and was dependent on nongovernmental organizations to conduct this critical response activity. The rapid deployment of healthcare providers to the scene of an outbreak could, as part of a broader health response, aid in achieving epidemic control by treating patients (thereby interrupting disease transmission), training local healthcare providers on infection control measures, and capturing and disseminating pertinent clinical observations and treatment protocols. Such an emergency medical team would be an asset domestically and internationally, in support of the WHO's Global Health Emergency Workforce.

In conclusion, it is impossible to know with certainty the location and nature of the next challenge to our national health security. However, it is safe to assume that one or more events that require a national-level response will occur in the near term. As a result, ensuring a high degree of public health preparedness should be a national priority.
